# Mediterranean Diet reduces ischemic heart disease risk in diabetes patients until reversed by smoking: Evidence from UK Biobank cohort

**DOI:** 10.1371/journal.pone.0336414

**Published:** 2025-11-14

**Authors:** Yongna Fan, Lihua Li, Fengjun Du, Jie Ren, Jing Dong, Bingyin Zhang, Xiaolei Guo, Yueqing Huang, Danru Liu, Jixiang Ma

**Affiliations:** 1 School of Public Health, Cheeloo College of Medicine, Shandong University, Jinan City, China; 2 People’s Hospital of Lixia District, Jinan City, China; 3 Department of Non-Communicable Disease Prevention, Shandong Province Center for Disease Control and Prevention, Jinan City, China; 4 Department of General Medicine, Suzhou Hospital of Nanjing Medical University/Suzhou Municipal Hospital, Suzhou City, China; 5 Shandong Provincial Chronic Disease Hospital (Shandong Provincial Rehabilitation Center), Qingdao City, China; Universiti Tunku Abdul Rahman Fakulti Perubatan dan Sains Kesihatan M Kandiah, MALAYSIA

## Abstract

**Objectives:**

This study aims to explore the association between the Mediterranean Diet (MD) pattern and the development of ischemic heart disease (IHD) in patients with diabetes mellitus (DM) to provide a scientific basis for the management of diabetic patients.

**Methods:**

Based on 7606 participants from the UK Biobank cohort, the adherence to the MD was assessed. The outcome indicator was the occurrence of IHD. Cox proportional hazards model was applied to estimate the Hazard ratios (HR) and 95% confidence interval (CI) of the Mediterranean Diet Score (MDS) and IHD, while trend analysis was performed using the Wald trend test. Subgroup and interaction analyses considered factors.

**Results:**

Among 7,606 diabetic patients with a mean follow-up of 13.04 years, a total of 1,173 new cases of IHD were reported. After multivariable adjustment, a higher MDS was negatively associated with the incidence of IHD in diabetic patients. Additionally, compared with the Q1 group, the Q4 group had a 18.5% lower risk of IHD occurrence. Trend tests were statistically significant (*P*-values < 0.001 for all four models). Subgroup analyses showed benefits in white people, males, high-income earners, non smokers and previous smokers, but a positive association in current smokers. Only gender, income, and smoking showing multiplicative interactions with MDS. Sensitivity analysis demonstrated effect modification by physical activity and education level, which was not observed in the primary analysis.

**Conclusions:**

The MD pattern can effectively reduce the incidence of IHD in diabetic patients, and gender, income and smoking have modifying effects on the MD pattern.

## Introduction

Diabetes Mellitus (DM) constitutes a major global public health challenge, affecting approximately 347 million individuals and imposing a growing burden on healthcare systems worldwide [1]. Current epidemiological data indicate a sharp upward trajectory in DM prevalence across all regions [2]. The principal clinical significance of DM lies in its induction of metabolic dysregulation, which manifests as multisystem complications—notably hypertension and dyslipidemia. These pathophysiological alterations act synergistically to accelerate atherosclerosis, culminating in a 2- to 4-fold elevation in cardiovascular disease (CVD) risk relative to non-diabetic populations. Among CVD outcomes, ischemic heart disease (IHD) represents one of the most severe DM complications, with diabetic patients facing twice the risk of IHD development compared to their non-diabetic counterparts [3].

Dietary intervention is recognized as a cornerstone strategy for mitigating cardiovascular sequelae in DM management [4,5]. Critically, human diets comprise complex combinations of foods and nutrients rather than isolated dietary components [6]. Furthermore, dietary effects are the result of the combined action of multiple dietary factors. Therefore, dietary patterns are recommended as a guideline for disease management.

The Mediterranean Diet (MD) is a globally recognized heart-protective dietary pattern characterized by a high intake of fruits, vegetables, legumes, whole grains, fish, and olive oil, as well as moderate consumption of red meat and red wine [7]. Multiple studies have confirmed that the MD can reduce the incidence and mortality risks of myocardial infarction [8],and coronary heart disease [9] in the general population. However, the cardioprotective effect of MD adherence against IHD incidence in diabetic populations remains unconfirmed, with critical knowledge gaps impeding precision dietary interventions—particularly concerning how behavioral factors (e.g., smoking, physical inactivity) and demographic characteristics modify this association.

Therefore, we relied on the UK Biobank prospective cohort to select 7,606 patients with DM at baseline and conducted an exploratory analysis of the association between MD and the incidence of IHD in DM patients, as well as the modifying effects of gender and other characteristics on this association. For DM patients, this study offers a foundation for precision dietary treatment.

## Materials and methods

### Study population

The UK Biobank(UKB) recruited more than 0.5 million participants aged 37–73 years from the general population between 2006 and 2010, and whose health status is being tracked, in part, through linked death and health records. The deadline for cohort recruitment for this study is November 30, 2022. Ethical approval for the UKB biospecimen bank study was obtained from the NHS North West Multicenter Research Ethics Committee (No. 21/NW/0157), and all participants signed a written informed consent form agreeing to join the study. Detailed information on study design, implementation, and data acquisition can be found at https://www.ukbiobank.ac.uk [10]. Participants were recruited at 22 assessment centers located throughout England, Wales, and Scotland [11]. At the assessment centre, the participants completed a touchscreen questionnaire, which collected information on socio-demographic characteristics and diet, lifestyle factors. Anthropometric measurements were taken using standardized procedures.

**Fig 1:**
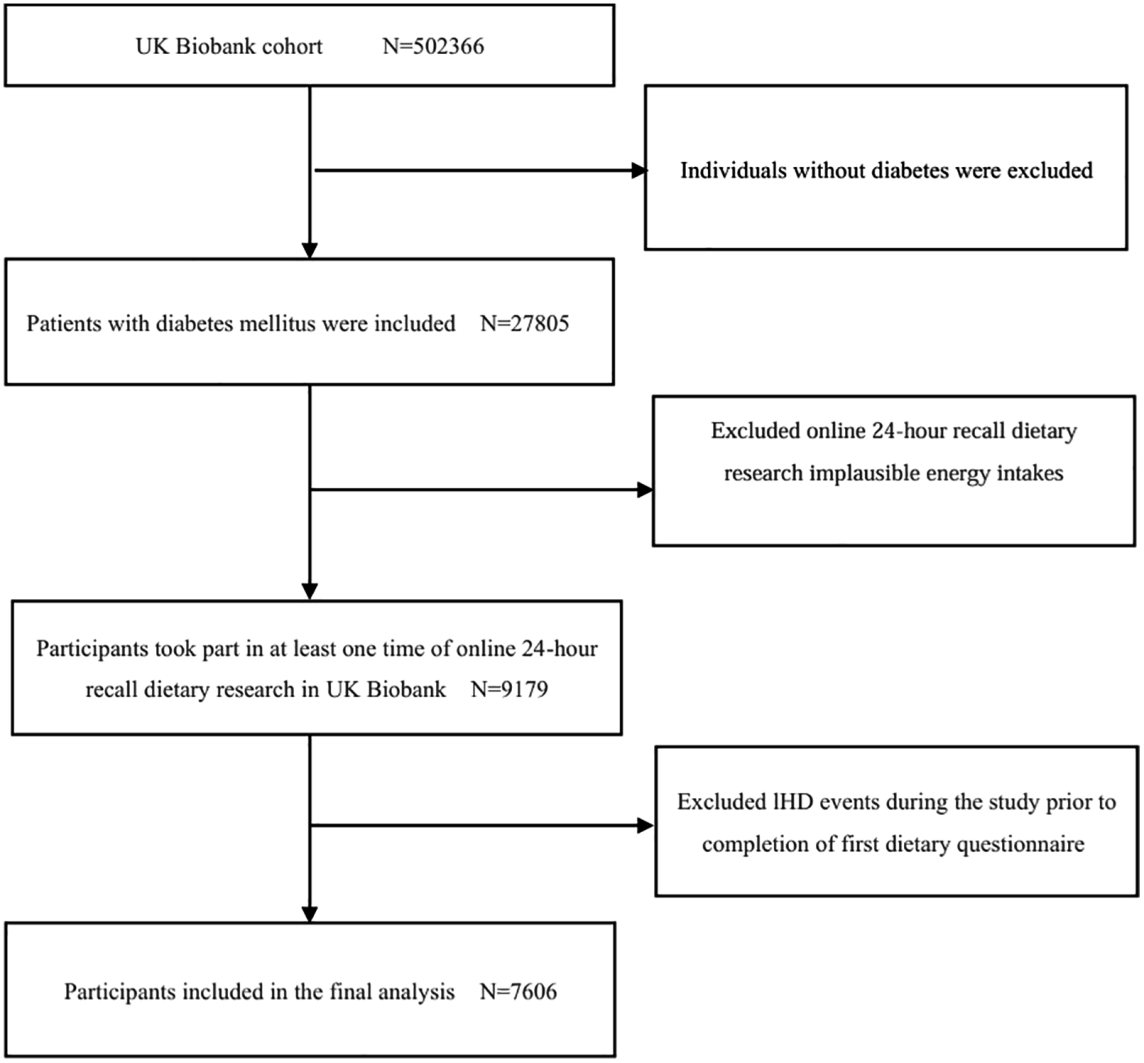
Selection of study participants in the UK Biobank.

This study utilized data from the UKB, including physician diagnoses, diabetes medication data, age at diagnosis of diabetes, date of first report (insulin-dependent diabetes mellitus, non-insulin-dependent diabetes mellitus, malnutrition-related diabetes mellitus, other specified diabetes mellitus, unspecified diabetes mellitus), self-reported data, and information coded according to the International Classification of Diseases, 10th Revision (ICD-10) and hospitalization dates, to identify individuals with diabetes. The corresponding ICD-10 codes for diabetes in this study are E10-E14, G590, G632, H280, H360, M142, and N083. In the initial cohort of 502,366 individuals, we included participants who had diabetes at baseline and had completed at least one 24-hour dietary survey, and excluded those with implausible total energy intake (men > 17,573 kJ or <3,347 kJ, women >14,644 kJ or <2,092 kJ). We further excluded participants who had IHD prior to their first 24-hour dietary assessment. Following these screening procedures, 7,606 persons were eventually selected for the research (Fig 1).

### Dietary assessment

In the UK Biobank cohort study, dietary information was collected using Oxford WebQ [12], an online self-administered 24-hour dietary assessment questionnaire specifically developed for large population studies. This dietary assessment requires participants to recall the frequency of consumption of 206 food items and 32 beverage items over the past 24 hours [13]. We calculated the quantity of each food or drink consumed by multiplying the assigned portion size of each food or beverage by the amount consumed. Food compositions from the UK Nutrient Databank were used to calculate nutrient intakes [12]. In the UKB follow-up cohort, five dietary questionnaires have been completed so far. To minimize the impact of within-population variability and random error, the average of ≥1 (≤5) 24-hour recall dietary assessments is used to calculate the dietary pattern index.

The Mediterranean Diet Score (MDS) is a dietary and nutritional-based scoring system designed to represent adherence to the MD. This study utilized the calculation method developed by Trichopoulou et al., which includes nine food groups, as it is the first and most widely used version of the MDS [14,15]. Foods are scored based on nine components: vegetables, legumes, fruits and nuts, grains, fish and seafood, the ratio of monounsaturated to saturated fats, dairy products, meat and meat products, and alcohol. We used gender-specific median intake as the cutoff point for each food group. Participants with intake levels above the median for vegetables, legumes, fruits and nuts, grains, fish and seafood, and the ratio of monounsaturated to saturated fats scored 1. Participants with intake levels below the median for dairy products, meat, and meat products scored 1. For alcohol, low to moderate intake (no more than 2 servings per day) scored 1. Non-drinkers or those consuming more than 2 servings per day scored 0. The total MDS ranged from 0 to 9, with higher scores indicating greater adherence to the MD [16]. An additional file shows this in more detail [S1 Table in S1 File].

### Follow-up and outcomes

The primary outcome measure of this study is the occurrence of IHD, as determined based on hospital admission records and death registry data related to the UK Biobank based on ICD-10 codes (I20-I25).

Participants were followed from their baseline visit until primary IHD diagnosis, death (through linkage with national death records), withdrawal from the study, or the end of follow-up which was November 30, 2022, whichever occurred first.

### Covariates

The following possible confounding factors were taken into account in this study: age (continuous: years), gender (male, female), ethnicity (white, non-white), educational level (Low: NVQ or HND or HNC or equivalent; Moderate: A levels/AS levels or equivalent, O levels/GCSEs or equivalent, CSEs or equivalent; High: College or University degree, other professional qualifications, e.g.,: nursing, teaching) [17], income(<£18,000,£18,000–£30,999,£31,000–£51,999,£52,000–£100,000, > £100,000), the Townsend Deprivation Index (continuous), smoking status (never, former, current), drinking frequency (daily or almost daily, 3–4 times per week, 1–2 times per week, 1–3 times per month, only on special occasions, never), physical activity (IPAQ: low, moderate, high), triglycerides (continuous), total cholesterol (continuous), low-density lipoprotein cholesterol (continuous), high-density lipoprotein cholesterol (continuous), family history of heart disease (yes, no), high blood pressure medication (yes, no), cholesterol medication (yes, no). In the subgroup analysis, age was further grouped into two categories: under 60 years old and 60 years old or older. An additional file shows this in more detail [S2 Table in S1 File].

### Statistical analysis

The qualitative data are presented as cases or percentages, and comparisons between the groups were made using the chi-squared test. Quantitative data are expressed as the mean ± standard deviation, with differences between groups assessed using the T-test.

This study utilized multiple imputation to impute covariates and conduct analyses. A multivariable Cox proportional hazards regression model was used to evaluate MDS as a continuous variable and as a categorical variable (grouped by quartiles) in order to investigate the relationship between MDS and the incidence of IHD in DM patients. Trend analysis was conducted using the Wald trend test. This study further conducted subgroup analyses based on baseline characteristics to explore the association between MDS and DM patients’ risk of IHD in different subgroups and to analyze the interaction between various factors and MDS. All models were adjusted for the following covariates: age, gender, ethnicity, education level, income, the Townsend Deprivation Index, smoking status, drinking frequency, physical activity, triglycerides, total cholesterol, high-density lipoprotein cholesterol, low-density lipoprotein cholesterol, family history of heart disease, high blood pressure medication, cholesterol medication. To reduce reverse causation, we conducted a sensitivity analysis and removed respondents with IHD in the first three years after enrollment to complete the primary analysis.

This study used R software (version 4.4.1) for statistical analysis. Two-sided tests were performed with a significance level of α = 0.05.

## Results

### Participant characteristics

A total of 7,606 patients with diabetes mellitus were included in the study, with an average age of (58.59 ± 7.29) years. Among them, 4,437 were male (58.3%) and 3,169 were female (41.7%). The median follow-up duration was 13.04 years, during which 1,173 patients (15.4%) developed IHD. The MDS for the general population was 3.98 ± 1.62, with a mean MDS score of 4.00 for non-IHD patients and 3.87 for IHD patients, a difference that was statistically significant (*P* = 0.009). The estimated triglyceride levels and age of IHD patients were greater. Compared to patients without IHD, participants with IHD tended to be males, with a family history of heart disease, using medication for high blood pressure or cholesterol, and with low education level. Specific characteristics are shown in Table 1.

**Table 1:** Basic characteristics of study participants.

Characteristic	Total Cases	Without IHD	IHD	*P* value^1^
Number of participants, N	7606	6433	1173	
MDS(mean(SD))	3.98(1.62)	4.00(1.62)	3.87(1.56)	**0.009**
Gender, N%				**<0.001**
Female	3169(41.7)	2809(43.7)	360(30.7)	
Male	4437(58.3)	3624(56.3)	813(69.3)	
Age(years) (mean (SD))	58.59(7.29)	58.25(7.36)	60.43(6.57)	**<0.001**
Ethnicity, N %				0.224
White	6790(89.7)	5757(89.9)	1033(88.7)	
Nonwhite	779(10.3)	647(10.1)	132(11.3)	
Income(pounds),N %				**<0.001**
Less than 18,000	1579(23.6)	1281(22.6)	298(29.0)	
18,000 - 30,999	1883(28.2)	1581(27.9)	302(29.4)	
31,000 - 51,999	1730(25.9)	1489(26.3)	241(23.4)	
52,000 - 100,000	1224(18.3)	1068(18.9)	156(15.2)	
More than 100,000	271(4.1)	240(4.2)	31(3.0)	
Townsend deprivation index(mean (SD))	−1.07(3.10)	−1.11(3.10)	−0.84(3.09)	**0.005**
Education level, N %				**0.001**
Low	507(7.8)	403(7.2)	104(10.9)	
Moderate	2118(32.5)	1820(32.7)	298(31.1)	
High	3892(59.7)	3336(60.0)	556(58.0)	
Smoking status, N %				**<0.001**
Never	3657(48.3)	3169(49.5)	488(41.9)	
Previous	3280(43.3)	2726(42.5)	554(47.6)	
Current	634(8.4)	512(8.0)	122(10.5)	
Alcohol intake frequency, N %				**0.03**
Daily or almost daily	1342(17.7)	1141(17.8)	201(17.1)	
Three or four times a week	1392(18.3)	1204(18.7)	188(16.0)	
Once or twice a week	1768(23.3)	1503(23.4)	265(22.6)	
One to three times a month	969(12.8)	822(12.8)	147(12.5)	
Special occasions only	1307(17.2)	1070(16.6)	237(20.2)	
Never	822(10.8)	687(10.7)	135(11.5)	
Physical activity level, N %				0.158
Low	1554(25.6)	1294(25.1)	260(28.1)	
Moderate	2503(41.2)	2135(41.4)	368(39.8)	
High	2020(33.2)	1723(33.4)	297(32.1)	
Triglycerides, mmol/L(mean(SD))	2.01(1.20)	1.98(1.17)	2.15(1.34)	**<0.001**
Total cholesterol, mmol/L(mean(SD))	4.62(1.08)	4.62(1.07)	4.60(1.10)	0.471
LDL cholesterol, mmol/L(mean(SD))	2.76(0.80)	2.76(0.80)	2.77(0.80)	0.748
HDL cholesterol, mmol/L(mean(SD))	1.26(0.35)	1.27(0.35)	1.20(0.35)	**<0.001**
Family history of heart disease, N%				**<0.001**
No	4139(54.4)	3572(55.5)	567(48.3)	
Yes	3467(45.6)	2861(44.5)	606(51.7)	
high blood pressure medication, N%				**<0.001**
No	3399 (44.7)	2979 (46.3)	420 (35.8)	
Yes	4203 (55.3)	3450 (53.7)	753 (64.2)	
cholesterol medication, N%				**<0.001**
No	2347 (30.9)	2046 (31.8)	301 (25.7)	
Yes	5255 (69.1)	4383 (68.2)	872 (74.3)	

Note: Continuous and categorical variables were expressed as mean (SD) and number (proportion).

Abbreviations: MDS, Mediterranean Diet Score; SD, standard deviation; N, number of participants; LDL cholesterol, low density lipoprotein cholesterol; HDL cholesterol, high density lipoprotein cholesterol. ^1^Obtained using the chi-squared test for the categorical variables and T-test for the continuous variables.

### Association between MDS and IHD incidence in patients with DM

The results showed that DM patients with high MDS had a lower risk of IHD events. In Model 1, the risk of IHD incidence was reduced by 5.5% (HR = 0.945, 95% CI: 0.930–0.960), and in Model 2, the risk of IHD incidence was reduced by 5.0% (HR = 0.950, 95% CI: 0.935–0.965), 3.7% reduction in the risk of IHD incidence in Model 3 (HR = 0.963, 95% CI: 0.947–0.979), and 3.5% reduction in the risk of IHD incidence in Model 4 (HR = 0.965, 95% CI: 0.949–0.981).

Compared to the lowest quartile, multivariable-adjusted HRs of IHD incidence in the third quartile and highest quartile were 0.916 (95% CI: 0.853–0.984)and 0.815(95% CI: 0.754–0.881), respectively. The trend test results were statistically significant (*P*-values for all four models were <0.001) (Table 2).

**Table 2:** COX regression analysis of MDS and IDH in patients with DM.

	Categorical				*P-*trend	continuous
	Q1	Q2	Q3	Q4		
Model1	Ref	**0.933 (0.874, 0.997)**	**0.851 (0.793, 0.914)**	**0.753 (0.697, 0.813)**	**<0.001**	**0.945 (0.930, 0.960)**
Model2	Ref	**0.917 (0.859, 0.979)**	**0.885 (0.824, 0.951)**	**0.765 (0.708, 0.826)**	**<0.001**	**0.950 (0.935, 0.965)**
Model3	Ref	0.940 (0.880, 1.004)	**0.917 (0.853, 0.985)**	**0.806 (0.745, 0.871)**	**<0.001**	**0.963 (0.947, 0.979)**
Model4	Ref	0.940 (0.880, 1.004)	**0.916 (0.853, 0.984)**	**0.815 (0.754, 0.881)**	**<0.001**	**0.965 (0.949, 0.981)**

Note: HR, hazard ratio;95% CI: 95% confidence interval; MDS, Mediterranean Diet Score; IQR: inter-quartile range; Q: quartile.

Model 1 is not adjusted. Model 2 was adjusted for age, gender, income, educational levels, Townsend deprivation index, ethnicity. Model 3 was further adjusted for smoking status, alcohol consumption frequency, physical activity level. Model 4 was further adjusted for triglycerides, total cholesterol, LDL cholesterol, HDL cholesterol, family history of heart disease, high blood pressure medication, cholesterol medication.

### Association between MDS and IHD incidence stratified by subgroups

**Fig 2:**
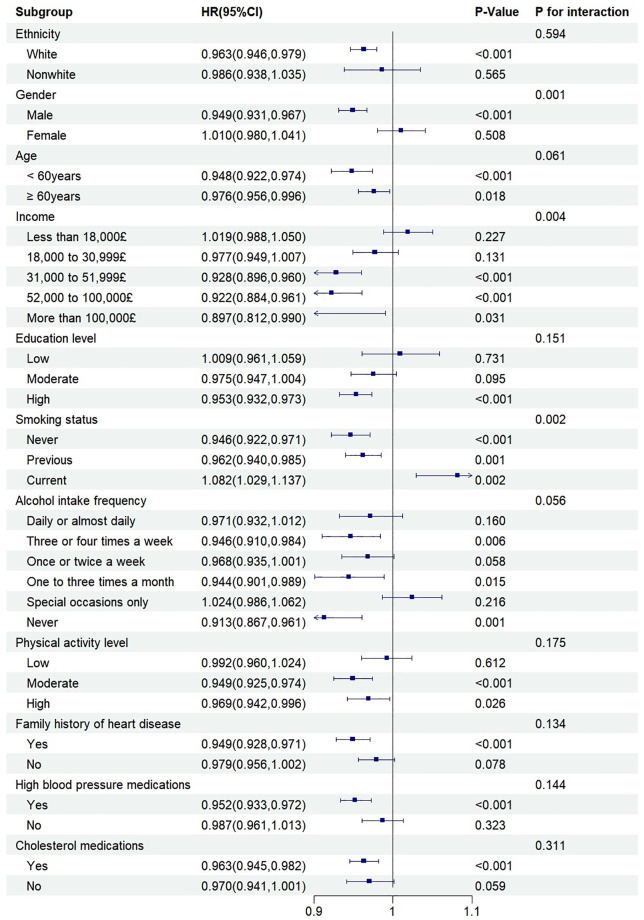
Subgroup analysis of MDS and IHD in DM patients. Note: HR, hazard ratio; 95% CI: 95% confidence interval. The model adjusted for age, gender, income, educational levels, Townsend deprivation index, ethnicity, smoking status, alcohol consumption frequency, physical activity level; triglycerides, total cholesterol, LDL cholesterol, HDL cholesterol, family history of heart disease, high blood pressure medication, cholesterol medication.

Subgroup analyses were conducted based on age, gender, ethnicity, education level, income, smoking status, drinking frequency, physical activity, family history of heart disease, cholesterol medication, and hypertension medication. The results showed that in subgroups of white individuals, males, those with an income of £31,000 or higher, never or former smokers, those with higher education levels, moderate or higher physical activity levels, a family history of heart disease, and use hypertension or cholesterol medication, MDS were negatively correlated with the incidence of IHD. However, among current smokers, higher MDS scores were associated with a higher risk of IHD incidence. Except for gender (interaction *P*-value = 0.001), income (interaction *P*-value = 0.004), and smoking (interaction *P*-value = 0.002), which had multiplicative interactions with MDS, other indicators did not have multiplicative interactions with MDS (*P* > 0.05) (Fig 2).

### Sensitivity analyses

Sensitivity analysis showed that adherence to the MD pattern had a protective effect among diabetic patients. Moreover, sex, income, and smoking status acted as effect modifiers for this relationship. Physical activity and education level also acted as effect modifiers.[S3 and S4 Tables in S1 File]

## Discussion

As is well known, patients with DM who develop IHD tend to have prolonged hospital stays, poor outcomes, and high mortality rates. It is anticipated that the individual and societal burden associated with DM and heart disease will continue to increase globally in the coming years [18]. Based on a prospective cohort study design, this study found that the MD has a significant cardiovascular protective effect on DM patients, meaning that DM patients with higher adherence to the MD diet have a significantly reduced risk of developing IHD. Notably, subgroup analysis revealed that the association between MDS and IHD exhibits population heterogeneity, with gender, high income level, and smoking status interacting to influence the incidence of IHD in DM patients.

The plant-based nutritional compose of the MD has been demonstrated to have substantial cardiovascular preventive effects, making it one of the most extensively researched dietary patterns in the world [19]. Although population-based cohort studies (such as the European Prospective Investigation into Cancer and Nutrition – Netherlands cohort) have revealed a significant negative correlation between MDS and the risk of IHD [20–24] (with a 30% reduction in myocardial infarction risk in the highest MDS group, and a 14% reduction in risk for every additional 2 MDS units) [25]. This study further extends this association to the DM patient population, where the highest MDS group showed only an 18.5% reduction in IHD incidence risk, lower than the effect of MDS on myocardial infarction in the general population. This disparity could be explained by either the severe food control requires for DM patients, which could lessen the dietary effect, or by the fact that DM patients have a markedly reduced vascular compensatory capacity, which would minimize the marginal effects of dietary intervention. It’s also important to note that the short follow-up period makes it difficult to evaluate long-term consequences like IHD precisely, leading to sparse and contradictory research findings that need more confirmation. In a prospective cohort study, 5,809 postmenopausal women with type 2 DM were recruited, and a higher adherence to the MD was associated with a 23% reduction in CVD risk [26]. However, this study did not include male patients or premenopausal women with DM, resulting in potential bias. In contrast, a cross-sectional study of DM patients found no significant association between the MD and cardiovascular risk factors [27]. The differing results may be due to the inability of cross-sectional studies to establish causal relationships between variables. According to a meta-analysis demonstrated that the MD plays a role in reducing the incidence and mortality of coronary heart disease in DM patients [28]. Consistent with the findings of this study, our results provide further evidence confirming that the MDS is a protective factor for DM patients, with higher MDS scores associated with lower IHD risk. However, Exclusion of early IHD cases in sensitivity analysis identified physical activity and education as effect modifiers in diabetes – consistent with prior evidence but contrasting with primary analysis, indicating probable reverse causation bias. Targeted research should resolve this discrepancy.

The above association may be attributed to the following mechanisms. First, MD includes substances such as fruits, vitamins, and legumes, which have significant antioxidant effects and can improve insulin sensitivity and pancreatic insulin secretion in DM patients, thereby reducing HbA1c levels and their impact on the vascular and endothelial systems in DM patients [29,30]. Additionally, the PREDIMED study reported that the MD can reduce risk factors such as blood pressure and lipid levels by decreasing inflammation and vascular endothelial damage, thereby lowering the risk of CVD in DM patients [31,32]. Meanwhile, a number of studies have demonstrated that the MD is a dietary pattern that can help people lose weight and avoid obesity [33].

Subgroup analysis showed that MDS was linked to a decreased risk of IHD in the subgroup with income ≥ £31,000, but not in the subgroup with income < £31,000, and that there was a multiplicative interaction between income and MDS. This is partially consistent with the findings of a cohort study conducted by Marialaura Bonaccio et al [34]. which observed that MD was associated with a lower risk of CVD, but this association was limited to higher socioeconomic groups. This may be attributed to the stronger ability of high-income groups to access healthy foods. Furthermore, this study discovered a multiplicative interaction between gender and MDS and revealed that MDS was significantly negatively linked with IHD in male DM patients but not in female patients. Delgado-Lista et al [35]. also observed similar gender-specific results. This may be because females typically possess less fat-free muscle mass compared to males, potentially exacerbating insulin resistance and dyslipidaemia [36]. Second, physiological differences related to reproductive factors may place females with diabetes at greater cardiometabolic risk. So the protective effect of increased MDS may have a smaller impact on women. Similarly, this study discovered that the protective impact of MDS on IHD in DM patients was significantly altered by smoking status. MDS and IHD were negatively correlated among never-smokers and ex-smokers, however there was an interaction effect and higher risk among current smokers. A possible explanation is that tobacco-derived free radicals synergistically exacerbate oxidative stress with hyperglycemia, offsetting the antioxidant effects of the diet. Further validation is needed, but this finding suggests that implementing a MD intervention to prevent IHD onset in DM patients should be contingent on smoking cessation.

This study has three major advantages: a prospective study design, a large sample size, and a long follow-up period, which help to avoid recall bias and reverse causality issues. Additionally, the MD pattern would help to control stable and comprehensive than individual foods.

However, this study also has some limitations. First, it is challenging to accurately quantify individual diets in large cohorts. The 24-hour recollection method, on which dietary assessments are based, has limitations in capturing long-term food patterns and may introduce measurement. However, we conducted multiple follow-up dietary surveys and calculated averages, which helped control for some measurement errors. Second, residual confounding from unmeasured or unknown factors may still remain even after we adjusted for the majority of confounding factors. Thirdly, given that most participants in the UK Biobank are white, this limits the generalizability of the study results. Finally, since we did not follow up on glycemic control, our current data cannot yet analyze the association between glycemic control and the cardioprotective effects of the MDS. Future research could utilize follow-up blood glucose data to explore this finding.

## Conclusions

In summary, the MD pattern has a significant effect on preventing the development of IHD in DM patients. Male, high-income individuals (≥£31,000), and those never-smokers or ex-smokers may derive additional benefits from the MD. Furthermore, implementing a MD intervention to prevent IHD development in DM patients should be contingent upon smoking cessation. This study provides scientific evidence for precision dietary interventions in DM patients.

## Supporting information

S1 File Supplementary Tables on the Mediterranean Diet and Risk of Ischemic Heart Disease in Diabetic Patients. This Appendix contains supplementary tables referenced in the paper, including food categories of the Mediterranean Diet pattern, data fields and definitions incorporating covariates, and results of Cox regression analysis and subgroup analysis examining the Mediterranean Diet pattern and ischemic heart disease in diabetes patients after excluding participants with ischemic heart disease within the first three years of follow-up.
(DOCX)
