# Supplementary material for: Mediterranean Diet reduces ischemic heart disease risk in diabetes patients until reversed by smoking: Evidence from UK Biobank cohort
Source: PLoS One. 2025 Nov 14;20(11):e0336414. doi: 10.1371/journal.pone.0336414 (PMC12617885; doi:10.1371/journal.pone.0336414)
Supplement: S1 File — This Appendix contains supplementary tables referenced in the paper, including food categories of the Mediterranean Diet pattern, data fields and definitions incorporating covariates, and results of Cox regression analysis and subgroup analysis examining the Mediterranean Diet pattern and ischemic heart disease in diabetes patients after excluding participants with ischemic heart disease within the first three years of follow-up. (DOCX) [file pone.0336414.s001.docx]

**Mediterranean diet reduces ischemic heart disease risk in diabetes until reversed by smoking: Evidence from UK Biobank cohort**

Authors: *Yongna Fan, Lihua Li , Fengjun Du, Jie Ren, Jing Dong, Bingyin Zhang, Xiaolei Guo, Yueqing Huang, Danru Liu, Jixiang Ma*

| **Page 2-3** | **S1 Table.** Components and scoring methods of the Mediterranean Diet Score (MDS) |
| --- | --- |
| **Page 4-6** | **S2 Table.** Data field and definition of the included covariates |
| **Page 7** | **S3 Table.** COX regression analysis of MDS and IDH in patients with DM, excluding participants with IHD within three years of baseline. |
| **Page 8-9** | **S4 Table.** Subgroup analysis of MDS and IHD in DM patients, excluding participants with IHD within three years of baseline. |

**S1 Table. Components and scoring methods of the Mediterranean Diet Score (MDS)**

|  | Indicator foods^1^ | Criteria for scoring |
| --- | --- | --- |
| 1.Vegetables (excluding potatoes, legumes or fruit juice) | Mixed vegetable, vegetable pieces, avocado, beetroot, broccoli, butternut squash, cabbage/kale, carrot, cauliflower, celery, courgette, cucumber, garlic, leek, lettuce, mushroom, onion, olives, parsnip, pea, side salad, sweet pepper, spinach Sprouts, sweetcorn, fresh tomato, tinned tomato, green bean, turnip/swede, watercress, other vegetables, homemade soup (vegetables) | Sex-specific median intakes used as cut points. Intakes (for indictors 1-6) above median score 1 and intakes below the median score 0. |
| 2. Legumes | Baked bean, pulses, broad bean, homemade soup (pulses) |  |
| 3. Fruit and nuts | Stewed fruit, prune, dried fruit, mixed fruit, apple, banana, berry, cherry, grapefruit, grape, mango, melon, orange, satsuma, peach/nectarine, pear intake, pineapple, plum, other fruit Orange juice, grapefruit juice, pure fruit/vegetable juice,Unsalted peanuts,unsalted nuts,types of spreads/sauces consumed (Peanut butter), seeds |  |
| 4. Cereals | Porridge, muesli, oat crunch, plain cereal, bran cereal, whole-wheat cereal, other cereal Bread consumed, sliced bread (mixed; wholemeal; seeded; other), baguette (mixed; wholemeal; seeded; other), bap (mixed; wholemeal; seeded; other), bread roll (mixed; wholemeal; seeded; other), other bread,White pasta, wholemeal pasta, white rice, brown rice, couscous, other grain Homemade soup, ingredients in homemade soup (pasta) |  |
| 5. Fish and seafood | Tinned tuna, oily fish, white fish, prawns, lobster/crab, shellfish, other fish,Homemade soup, ingredients in homemade soup (fish) |  |
| 6.Monounsaturated/ saturated fats ratio | Monounsaturated fats, saturated fats |  |
| 7. Dairy products | Milk, milk added to cereal,Low fat hard cheese, low fat cheese spread, cottage cheese,Yogurt (low fat yogurt consumer; full fat yogurt consumer), Goat's cheese, hard cheese, soft cheese, blue cheese, cheese spread, feta, mozzarella, other cheese ,Dairy smoothie, latte, added milk to instant coffee, added milk to filtered coffee, added milk to espresso, added milk to other coffee type, added milk to standard tea, added milk to rooibos tea, cappuccino | Sex-specific median intakes used as cut points. Intakes (for indictors 7-8) below median score 1 and intakes above the median score 0. |
| 8. Meat and meat products | Beef, pork, lamb, other meat, Whole egg, omelette, eggs in sandwiches, scotch egg, other egg, Homemade soup, ingredients in homemade soup (meat), sausage, bacon, ham |  |
| 9. Alcohol | Red wine, rose wine, white wine, Beer/cider Fortified wine, spirits intake, other alcohol | No more than 2 drinks/day = 1; Never drink or over 2 drinks/day = 0. |

Note：^1^As available in the UK Biobank

**S2 Table. Data field and definition of the included covariates.**

| **Covariates** | **Data field** | **Description** | **category** |
| --- | --- | --- | --- |
| Age | 21003 | Age at first recruitment | continuous, years |
| Sex | 31 | Self-reported sex during the initial Assessment Centre visit | \| 0 \| Female \| \| --- \| --- \| \| 1 \| Male \| |
| Ethnicity | 21000 | Self-reported ethnic background during the initial Assessment Centre visit | - |
| Educational level | 6138 | ACE touchscreen question "Which of the following qualifications do you have? (You can select more than one)" | \| 1 \| College or University degree \| \| --- \| --- \| \| 2 \| A levels/AS levels or equivalent \| \| 3 \| O levels/GCSEs or equivalent \| \| 4 \| CSEs or equivalent \| \| 5 \| NVQ or HND or HNC or equivalent \| \| 6 \| Other professional qualifications eg: nursing, teaching \| \| -7 \| None of the above \| \| -3 \| Prefer not to answer \| |
| Income | 738 | ACE touchscreen question "What is the average total income before tax received by your HOUSEHOLD?" | 1 Less than 18,000  2 18,000 to 30,999  3 31,000 to 51,999  4 52,000 to 100,000  5 Greater than 100,000  -1 Do not know  -3 Prefer not to answer |
| Townsend deprivation index | 22189 | Townsend deprivation index calculated immediately prior to participant joining UK Biobank. Based on the preceding national census output areas. Each participant is assigned a score corresponding to the output area in which their postcode is located. | continuous |
| Alcohol intake frequency | 1558 | ACE touchscreen question "About how often do you drink alcohol?" | \| 1 \| Daily or almost daily \| \| --- \| --- \| \| 2 \| Three or four times a week \| \| 3 \| Once or twice a week \| \| 4 \| One to three times a month \| \| 5 \| Special occasions only \| \| 6 \| Never \| \| -3 \| Prefer not to answer \| |
| Smoking status | 20116 | This field summarises the current/past smoking status of the participant. | -3 Prefer not to answer  0 Never  1 Previous  2 Current |
| Physical activity | 22032 | UK Biobank Touchscreen questionnaire at baseline | 0 low  1 moderate  2 high |
| triglycerides | 30870 | Measured by GPO-POD analysis on a Beckman Coulter AU5800 | continuous, mmol/L |
| total cholesterol | 30690 | Measured by CHO-POD analysis on a Beckman Coulter AU5800 | continuous, mmol/L |
| LDL cholesterol | 30780 | Measured by enzymatic protective selection analysis on a Beckman Coulter AU5800 | continuous, mmol/L |
| HDL cholesterol | 30760 | Measured by enzyme immunoinhibition analysis on a Beckman Coulter AU5800 | continuous, mmol/L |
| family history of heart disease | 20107;  20110;  20111 | This field contains the combined results of 2 ACE touchscreen questions, both asked "Has/did your father ever suffer from? (You can select more than one answer)"  "Has/did your mother ever suffer from? (You can select more than one answer)"  "Have any of your brothers or sisters suffered from any of the following diseases? (You can select more than one answer)" | - |
| High blood pressure medication | 6177；  6153 | ACE touchscreen question "Do you regularly take any of the following medications? (you can select more than one answer)" |  |
| cholesterol medication | 6177；  6153 | ACE touchscreen question "Do you regularly take any of the following medications? (you can select more than one answer)" |  |

**S3 Table. COX regression analysis of MDS and IDH in patients with DM, excluding participants with IHD within three years of baseline.**

|  | Categorical | | | | | | |  | *P-*trend |  | continuous |
| --- | --- | --- | --- | --- | --- | --- | --- | --- | --- | --- | --- |
|  | Q1 |  | Q2 |  | Q3 |  | Q4 |  |  |  |  |
| Model1 | Ref |  | 0.967（0.903, 1.036） |  | **0.878（0.815, 0.946）** |  | **0.768（0.709, 0.833）** |  | **<0.001** |  | **0.950（0.934, 0.966）** |
| Model2 | Ref |  | 0.948（0.885, 1.016） |  | **0.908（0.842, 0.978）** |  | **0.776（0.715, 0.841）** |  | **<0.001** |  | **0.953（0.937, 0.970）** |
| Model3 | Ref |  | 0.973（0.908, 1.042） |  | 0.941（0.873, 1.014） |  | **0.818（0.754, 0.888）** |  | **<0.001** |  | **0.967（0.950, 0.983）** |
| Model4 | Ref |  | 0.973（0.908, 1.043） |  | 0.939（0.871, 1.013） |  | **0.826（0.761, 0.897）** |  | **<0.001** |  | **0.969（0.952, 0.985）** |

Note：HR, hazard ratio;95% CI: 95% confidence interval; MDS, mediterranean diet score; IQR: inter-quartile range；Q: quartile.

Model 1 is not adjusted. Model 2 was adjusted for age, gender, income, educational levels, Townsend deprivation index, ethnicity. Model 3 was further adjusted for smoking status, alcohol consumption frequency, physical activity level. Model 4 was further adjusted for triglycerides, total cholesterol, LDL cholesterol, HDL cholesterol, family history of heart disease, high blood pressure medication, cholesterol medication.

**S4 Table. Subgroup analysis of MDS and IHD in DM patients, excluding participants with IHD within three years of baseline.**

| **Subgroup** | **HR(95%CI)** | ***P*-value** | ***P* for interaction** |
| --- | --- | --- | --- |
| **Ethnicity** |  |  | 0.607 |
| White | 1.000 | **-** |  |
| Nonwhite | 1.014(0.963,1.068) | 0.606 |  |
| **Gender** |  |  | **0.005** |
| Female | 1.000 | **-** |  |
| Male | **0.949(0.915, 0.984)** | **0.005** |  |
| **Age** |  |  | 0.703 |
| < 60years | 1.000 | **-** |  |
| ≥ 60years | 1.007 (0.972,1.042) | 0.703 |  |
| **Income** |  |  | **0.017** |
| Less than 18,000£ | 1.000 | - |  |
| 18,000 to 30,999£ | 0.969 (0.928,1.014) | 0.173 |  |
| 31,000 to 51,999£ | **0.931 (0.888,0.977)** | **0.003** |  |
| 52,000 to 100,000£ | **0.922 (0.875,0.973)** | **0.003** |  |
| More than 100,000£ | 0.981 (0.885,1.087) | 0.710 |  |
| **Education level** |  |  | **0.024** |
| Low | 1.000 | - |  |
| Moderate | 1.004 (0.947,1.064) | 0.903 |  |
| High | **0.945 (0.896,0.998)** | **0.041** |  |
| **Smoking status** |  |  | **<0.001** |
| Never | 1.000 | - |  |
| Previous | 1.012 (0.976,1.049) | 0.522 |  |
| Current | **1.136 (1.073,1.203)** | **<0.001** |  |
| **Alcohol intake frequency** |  |  | 0.096 |
| Daily or almost daily | 1.000 | - |  |
| Three or four times a week | 0.984 (0.928,1.043) | 0.577 |  |
| Once or twice a week | 1.013 (0.959,1.070) | 0.652 |  |
| One to three times a month | 1.003 (0.941,1.068) | 0.932 |  |
| Special occasions only | **1.059 (1.002,1.121)** | **0.043** |  |
| Never | 0.984 (0.920,1.052) | 0.634 |  |
| **Physical activity level** |  |  | **0.018** |
| Low | 1.000 | - |  |
| Moderate | **0.949(0.910,0.991)** | 0.016 |  |
| High | **0.941(0.901,0.984)** | 0.007 |  |
| **Family history of heart disease** |  |  | 0.599 |
| Yes | 1.000 | - |  |
| No | 1.009(0.976,1.044) | 0.599 |  |
| **High blood pressure medications** |  |  | 0.316 |
| Yes | 1.000 | - |  |
| No | 1.018(0.983,1.054) | 0.316 |  |
| **Cholesterol medications** |  |  | 0.403 |
| Yes | 1.000 | - |  |
| No | 1.016(0.979,1.055) | 0.403 |  |

Note：HR, hazard ratio;95% CI: 95% confidence interval.

The model adjusted for: age, gender, income, educational levels, Townsend deprivation index, ethnicity, smoking status, alcohol consumption frequency, physical activity level; triglycerides, total cholesterol, LDL cholesterol, HDL cholesterol, family history of heart disease, high blood pressure medication, cholesterol medication.
